# IAP antagonist GDC-0917 is more potent than Debio1143 in promoting cell death, c-IAP1 degradation and tumor growth inhibition

**DOI:** 10.1038/s41419-022-05283-w

**Published:** 2022-09-28

**Authors:** Bruno Alicke, Eugene Varfolomeev, Shi Hui Kaylee Lee, Alexandra Frommlet, Savita Ubhayakar, John G. Quinn, Wayne J. Fairbrother, Robert Jones, Stephen E. Gould, Domagoj Vucic

**Affiliations:** 1 Departments of In Vivo Pharmacology, Genentech, South San Francisco, CA 94110 USA; 2 Immunology Discovery, Genentech, South San Francisco, CA 94110 USA; 3 Early Discovery Biochemistry, Genentech, South San Francisco, CA 94110 USA; 4 Drug Metabolism and Pharmacokinetics, Genentech, South San Francisco, CA 94110 USA; 5 Biochemical and Cellular Pharmacology, Genentech, South San Francisco, CA 94110 USA

**Keywords:** Cancer models, Apoptosis

Proper regulation of cell death and survival is essential for cellular homeostasis. Inhibitor of apoptosis (IAP) proteins play pivotal roles in cellular survival by blocking cell death, modulating signal transduction, and affecting cellular proliferation. By contributing to the evasion of cell death, IAP proteins enhance the resistance of cancer cells to treatment with antitumor agents and impact tumor progression. IAP proteins can be negated by SMAC (second mitochondrial activator of caspases) and SMAC-mimicking IAP antagonists [1]. IAP antagonism results in the activation of noncanonical NF-kB signaling and TNF-mediated cell death [2–4]. A number of IAP antagonists have been tested in clinic with overall favorable pharmacological properties, for example, GDC-0917 [1, 5], and Debio1143 (SM-406, AT-406, xevinapant) [6–8]. It was recently reported that IAP antagonist Debio1143 exhibits an 18-fold higher concentration in tumors than in plasma of cancer patients, which could lead to an inherently higher therapeutic index [9]. Here, we compare the cell death-inducing and anti-tumor capabilities of GDC-0917 and Debio1143, and investigate their tumor and plasma concentrations in two preclinical mouse tumor models. We find that GDC-0917 is more potent in cells and in vivo compared to Debio1143 and could not verify the tumor-selective tissue distribution previously reported albeit in non-clinical systems. Thus, we conclude that c-IAP1 degradation and cell death induction are better predictors of the potential application of IAP antagonists in the clinical setting than tumor to plasma exposure ratios.

Using surface plasmon resonance (SPR), we found that IAP antagonists GDC-0917 and Debio1143 preferentially bind BIR3 domains over BIR2 domains of c-IAP1 and XIAP (Fig. S1A, B) [10]. To investigate the cellular activity of GDC-0917 and Debio1143, we treated several cancer cell lines with increasing amounts of each compound. Both GDC-0917 and Debio1143 induced death in MDA-MB-231, A2058, EVSA T and EFM192A cells but GDC-0917 was more efficient and showed the ability to trigger cell death at 10-100 fold lower concentrations compared to Debio1143 (Fig. 1A and Fig. S1C). These two IAP antagonists also promoted c-IAP1 degradation and p100 processing to p52 (an indication of the activation of noncanonical NF-kB signaling) with GDC-0917 again showing higher potency (Fig. 1B and Fig. S1D) [11].

**Fig. 1:**
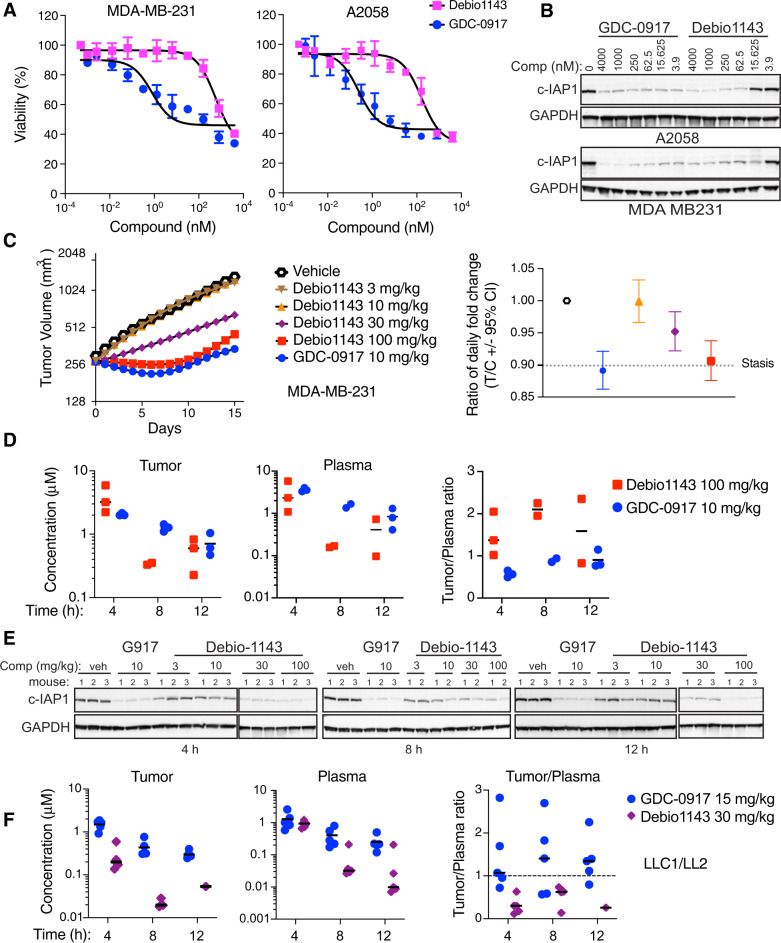
GDC-0917 is more efficient than Debio1143 in inducing death of cancer cells, c-IAP1 degradation and inhibition of tumor growth. **A** IAP antagonists GDC-0917 and Debio1143 induce cell death. MDA-MB-231 and A2058 cells were treated with increasing amounts of GDC-0917 and Debio1143 for 20 h. Cell viability was determined as described in Experimental procedures. **B** c-IAP1 degradation by GDC-0917 and Debio1143 is dose dependent. A2058 and MDA-MB-231 cells were treated with indicated concentrations of GDC-0917 and Debio1143 for 1 h and cellular lysates were examined by western blotting using antibodies against cIAP1. GAPDH expression levels were used as control. **C** Tumor-inhibiting activity of GDC-0917 and Debio1143 in vivo. B-17 SCID.bg mice bearing similarly sized subcutaneous MDA-MB-231-X1.1 tumors were separated into groups and treated daily with vehicle, GDC-0917, or Debio1143 via oral gavage. Tumor volume measurements (Left panel) and analysis were performed as described in Experimental Procedures. Group level-statistical comparisons (Right panel) were performed based on the ratio of daily fold change for the treatment (T) and vehicle control (C) groups over the duration of the study (stasis = 0.89). **D** Levels of GDC-0917 and Debio1143 (does at 100 mg/kg) in tumors and plasma from the study described in **C**. **E** GDC-0917 is more efficient compared to Debio1143 in promoting c-IAP1 degradation in tumor tissues. MDA-MB-231-X1.1 xenografts from mice treated with GDC-0917 (G917), Debio1143 or vehicle once daily for 16 days as indicated above were collected 4, 8 or 12 h after completion of the dosing period. Tumor tissue lysates were prepared as described in Experimental procedures. Expression of c-IAP1 was examined by western blotting with the indicated antibody and with GAPDH as a loading control; one of two repeats shown. **F** Levels of GDC-0917 and Debio1143 in tumors and plasma from mice bearing subcutaneous LLC1/LL2 tumors. Experiments in A and B were repeated three times, and in **C**–**F** once with the following number of mice: 9 for the vehicle and GDC-0917 groups, and 8 per dose arm of Debio1143 in **C**; 15 mice per compound with 5 mice per time point in were used in **F**.

We next wanted to compare the activity of GDC-0917 and Debio1143 in vivo and evaluated the relative activity of both drugs in the MDA-MB-231 xenograft model. We chose a dose of 10 mg/kg for GDC-0917, since it was a near maximally effective dose but also a well-tolerated dose based on prior work [12]. We tested a range of doses of Debio1143, based on published reports, dosing up to 100 mg/kg [13]. Remarkably a 10 mg/kg dose of GDC-0917 was comparable or even slightly more efficient in inhibiting tumor growth than a 10-fold higher dose of Debio1143 (Fig. 1C and Fig. S2A) [14]. As a recent study indicated that Debio1143 greatly accumulates in tumor tissues [9], we assayed both tumor and plasma concentrations for both drugs at 4, 8, and 12 h following the last dose in this study. Overall plasma and tumor exposure of GDC-0917 at 10 mg/kg was similar to the exposure of Debio1143 dosed at 100 mg/kg (Fig. 1D and Fig. S2B). Debio1143 concentrations showed comparable or a slightly higher tumor to plasma (T/P) ratio which increased with time, likely driven by lower levels of Debio1143 compound detected in plasma at later time points (Fig. 1D and Fig. S2B). Importantly, GDC-0917 was more efficient in promoting c-IAP1 degradation in MDA-MB-231 tumors and in the livers of tumor-bearing mice (Fig. 1E and Fig. S3A).

In order to extend these observations to a second model, we dosed mice bearing subcutaneous LL/2 (LCC1) tumors with 15 mg/kg GDC-0917 or 30 mg/kg Debio1143 (the dose of Debio1143 that showed the highest tumor to plasma ratio in the MDA-MB-231 model). Analyses of both drugs over several time points revealed similar exposures for both drugs in plasma but with GDC-0917 exhibiting slightly higher T/P ratios (Fig. 1F). Similar to the observation in the MDA-MB-231 model, examination of LL/2 (LCC1) tumor tissues showed that GDC-0917 was more efficient in causing c-IAP1/2 degradation (Fig. S3B).

Overall, these findings indicate that GDC-0917 has better potency in stimulating the death of cancer cells, promoting c-IAP1 degradation, and in inhibiting tumor growth than Debio1143. Importantly, neither molecule appeared to accumulate extensively or consistently within the tumor with in vitro potency and in vivo pharmacodynamics being better predictors of in vivo antitumor activity than T/P ratio. A number of factors may contribute to the observed higher potency of GDC-0917 compared to Debio1143 (for example, tighter XIAP BIR3 binding or more potent c-IAP1 degradation). We trust that this study will help in the future evaluation of IAP antagonists in hopes of bringing these promising reagents to cancer patients who need them most.

## Supplementary information

Supplemental Material

## Data Availability

All the data and reagents published in this study are available upon request.
