# Supplementary material for: IAP antagonist GDC-0917 is more potent than Debio1143 in promoting cell death, c-IAP1 degradation and tumor growth inhibition
Source: Cell Death Dis. 2022 Sep 28;13(9):831. doi: 10.1038/s41419-022-05283-w (PMC9519744; doi:10.1038/s41419-022-05283-w)

IAP antagonist GDC-0917 is more potent than Debio1143 in promoting cell death, c-IAP1 degradation and tumor growth inhibition

Bruno Alicke[1,*], Eugene Varfolomeev[2,3,*], Shi Hui Kaylee Lee[4], Alexandra Frommlet[5], Savita Ubhayakar[4], John G. Quinn[5], Wayne J Fairbrother[3], Robert Jones[4], Stephen E. Gould[1], Domagoj Vucic[2,3,#]

Supplementary information

Experimental procedures

Cell Lines and Reagents

A2058 human melanoma, MDA-MB-231 human breast carcinoma, and LL/2 (LLC1) murine lung carcinoma cells were obtained from ATCC. EVSA-T and EFM-192A human breast carcinoma cells were obtained from DSMZ. All cell lines were grown in 50:50 Dulbecco's modified Eagle's and FK12 medium supplemented with 10% FBS, penicillin and streptomycin. GDC-0917, Debio1143 and reserpine were synthetized at Genentech. A generic internal standard labetalol was purchased from Sigma Aldrich (St. Louis, Missouri). HPLC-grade water, HPLC-grade acetonitrile, and formic acid were purchased from Sigma Aldrich (St. Louis, Missouri). Shallow injection plates (350 µL) were purchased from Analytical Sales and Service (Pompton Plains, New Jersey).

Surface Plasmon Resonance

SPR experiments were performed on a Biacore S200 instrument (Cytiva). Proteins were immobilized to 400-600 RU through the His-tag to a Cytiva Nickel-NTA chip. Flow cell 1 was used as the reference channel (no protein immobilized).The running buffer contained 50mM HEPES, pH 7.2, 150 mM NaCl, 0.2%PEG-3350, 0.5mM TCEP, and 2% DMSO when compounds were present. The compounds were injected in a 8-point titration, with a 2-fold dilution and concentration ranging from 1.56nM to 200nM for c-IAP1 BIR3 and XIAP BIR3 binding, or 78.1nM to 10uM for c-IAP1 BIR2 and XIAP BIR2 binding. Production and purification of recombinant c-IAP1 and XIAP BIR2 and BIR3 proteins was previously described [12]. The association time was 45s, the

dissociation time varied between 60s and 1200s. Data was acquired using either Multi-Cycle Kinetic or, for very potent compounds, using Single-Cycle Kinetic. All data was analyzed using Biacore S200 Evaluation Software (1.1, Cytiva). All data were blank subtracted and a solvent correction was applied.

Tissue protein lysis preparation

Tumor and liver tissues samples were lysed in 10-fold tissue weight-volume (ml/mg) of cold Tissue Lysis Buffer (TLB): 20 mM Tris–HCl, pH 7.5, 150 mM NaCl, 4 mM EDTA, 1% Triton X-100, 0.1 % SDS supplemented with protease and phosphatase inhibitor cocktail (Thermo Fisher Scientific) followed by extraction with Bead Raptor Homogenizer (OMNI International, Kennesaw, GA, USA) according to manufactures instructions. Lysates were spun at 20000 g for 10 minutes at 4°C. Protein concentrations were determined by BCA assay (Thermo Fisher, Waltham, MA, USA). About 30 mg of protein lysates were used to analyze expression of the proteins by western blotting as indicated in the figure legends.

For analysis of c-IAP1/2 and p100 processing expression in cancer cells protein samples, 4 x 10^5 A2058 and MDA-MB-231 cells were seeded into 6 cm^2 cultured dishes. Cells were treated as indicated in the figure legends. 5 μM emricasan was added to the treatment media in the case of p100 processing analysis. Protein sample lysates were prepared as describe in [13]. Expression of human c-IAP1 or combined c-IAP1/2 expression in cancer cell protein lysates or tissue lysates was examined by western blotting as described previously [13]. The primary antibodies against human c-IAP1 (#AF8181), c-IAP1/2 pan specific antibodies (#MAB3400) were purchased from R&D, anti-p100/p52 antibody (#05-361) was from Millipore, anti GAPDH (#2118) from Cell Signaling Technology.

Viability Assays

Cells (1–1.5 × 10^4 per well) were seeded into 96-well dishes. Cells were treated as indicated in the figure legends. Cell viability was measured by CellTiter-Glo assay (Promega) according to experimental procedures.

Tumor xenograft studies

All procedures involving animals were performed in accordance with Genentech's Institutional Animal Care and Use Committee guidelines. *In vivo* studies were conducted using two cell lines maintained *in vitro*: MDA-MB-231 X1.1, an in-house in vivo-selected human breast adenocarcinoma cell line engineered to express GFP, which was derived from the MDA-MB-231 cell line obtained from American Type Culture Collection (ATCC) (Manassas, VA) and LL/2 (LLC1), a murine lung carcinoma cell line (ATCC). Both cell lines were cultured in RPMI 1640 media + 1% L-glutamine with 10% fetal bovine serum. For inoculation into animals, cells were trypsinized using 1X Trypsin 0.5% with ethylenediaminetetraacetic acid (EDTA) in phosphate-buffered saline (PBS) and collected in RPMI 1640 media + 1% L-glutamine with 10% fetal bovine serum. Cells were then centrifuged, washed once with Hanks' Balanced Salt Solution (HBSS), counted, and resuspended in HBSS and Matrigel (Corning catalog 356237) at a ratio of 1:1 (v:v) at a concentration of 100 and 1 million cells/mL, respectively. MDA-MB-231-X1.1 tumors were generated by inoculating 21-week old female C.B-17 SCID.bg mice (Charles River Labs, Hollister, CA) subcutaneously with 10×10^6 cells into the right thoracic flank in 0.1 ml. On Day 0, when tumors reached a size of 161-384 mm^3 (279 ± 7 mm^3, mean $\pm$ SD), administration of GDC-0917 and Debio1143 once a day for 16 consecutive days was initiated. Both molecules were formulated in 15% hydroxypropyl-beta-cyclodextrin and 20 mM succinic acid and administered orally by gavage in a volume of 10 mL/kg at the dose levels indicated elsewhere. Plasma and tumor were collected at 4, 8, 12 hours after the final administration to conclude the 16 day dosing period. Subcutaneous LL/2 (LLC1) tumors were generated similarly in 14-week old female C57Bl6 mice (Charles River Labs, Hollister, CA) with 0.1×10^6 cells. Once tumors were palpable, GDC-0917 and Debio1143 were prepared and administered once as described above. Plasma and tumor were collected 4, 8, 12 hours thereafter.

Tumor volumes were measured in 2 perpendicular dimensions (length and width) using calipers. Tumor volume was calculated using the following formula: Tumor size (mm^3) = length $\times$ width^2 $\times$ 0.5.

Analyses and comparisons of tumor growth were performed using a package of customized functions in R (Version 4.1.0; R Foundation for Statistical Computing; Vienna, Austria), which integrates software from open source packages (e.g., lme4, mgcv, gamm4, multcomp, settings, and plyr) and several packages from tidyverse (e.g., magrittr, dplyr, tidyr, and ggplot2) as described previously [14]. Ratios of daily fold change equal to <1 indicate an antitumor effect; the smaller the value below 1, the greater the magnitude of the antitumor effect. A ratio of 1 is indicative of no treatment effect (i.e., the daily fold changes are equivalent in both groups). Values in parentheses indicate the upper and lower boundaries of the 95% confidence interval for the difference based on the fitted model and variability measures of the data.

Body weights were also fitted with a generalized additive mixed model (Forrest et al. 2020), The raw body weight data at each timepoint from all individual animals and group fits were normalized to the starting weight and reported as percent body weight change.

Bioanalysis of GDC-0917 and Debio1143

Instrumentation and Chromatographic Conditions:

A Nexera UPLC system (Shimadzu, Kyoto, Japan) including a Shimadzu SIL-30AD solvent delivery system, a SIL-30AC autosampler and a CTO-30AC column oven were used to analyze GDC-0917 and Debio1143 in all three matrices (plasma, liver and tumor). Chromatographic separation was achieved by using a Phenomenex XB-C18 column (50 x 2.1 mm, 2.7 µm) with gradient elution using 0.1% formic acid in water and 0.1% formic acid in acetonitrile. The LC flow rate was 1.1 mL/min for both compounds. The sample injection volume was 5 µL and the retention times were 0.62 minutes for both compounds as well as for the internal standard (labetalol).

Mass Spectrometry:

A QTrap®5500 tandem mass spectrometer (Sciex, Foster City, CA) with Turboionspray (TIS) interface was operated in positive ionization mode with multiple reaction monitoring (MRM) for LC-MS/MS analysis. The optimized instrument parameters for monitoring these five compounds were as follows: TIS temperature: 500ºC; TIS voltage: 5500 V; curtain gas (CUR): 20; nebulizing gas (GS1): 50, Heater gas (GS2): 50, and collision gas: medium. The precursor to product ion transitions and other detailed

parameters are summarized in Table 1. The mass spectrometer was operated at unit mass resolution for both Q1 and Q3 quadrupoles.

Sample Preparation:

Both GDC-0917 and Debio1143 were prepared at 1 mg/ml in 100% DMSO for the stock solution. A working standard spiking solution of 0.1 mg/ml in 100% DMSO was also prepared. The ISTD spiking solution (200 ng/ml) was combined with 100% acetonitrile to suffice as both the internal standard as well as the protein precipitation solvent. Serially-diluted calibration curve-spiking solutions were made in 100% DMSO with the calibration curve points ranging from 20,000 ng/ml to 1 ng/ml. Prior to sample extraction, livers and tumors were first homogenized using pure water for a total dilution factor of 5. Calibration curves were then prepared using commercially purchased blank matrix (plasma, liver homogenate and tumor homogenate). Briefly, 25 µL of samples (plasma and tissue homogenates) were pipetted out into individual cluster tubes. Appropriate amounts of standard working solutions (5 µL) were spiked into 25 µL of blank matrix to generate a calibration curve ranging from 1 ng/ml to 20,000 ng/ml. 125 µL of internal standard-spiking solution/ACN (200 ng/ml) were added to all samples and calibration curves. Samples were then vortexed for 10 minutes and centrifuged at 3700 rpm for 10 minutes at 4ºC. After centrifugation, 50 µL of supernatant were transferred to a 350 µL shallow-well injection plate containing 150 µL water and placed in the autosampler for injection.

Table 1. Mass Spectrometry Parameters.

Compound	Q1/Q3	DP	CE	CXP
GDC-0917	565/341	16	29	22
Debio1143	562/167	31	35	12
Labetalol (ISTD)	329/294	101	35	10

Supplementary Figures

Supplementary Figure 1. GDC-0917 and Debio1143 promote cell death, c-IAP1 loss and activation of noncanonical NF-kB signaling.

A and B. GDC -017 and Debio1143 preferentially bind BIR3 domains of c-IAP1 and XIAP. SPR assays were performed using recombinant BIR2 and BIR3 domains of XIAP and c-IAP1 with indicated compounds (A). Proteins were immobilized on an SPR chip and compounds were injected at concentrations ranging from 1.56 nM to 200 nM for BIR3 domains binding, or 78.1 nM to 10 μM for BIR2 domains binding (B). The KD of GDC-0917 on XIAP BIR3 could not be determined because its low dissociation rate constant produced a dissociation response that was not resolvable from baseline drift. The affinity is thus reported only as estimated to be less than 0.80 nM.

C. IAP antagonists GDC-0917 and Debio1143 induce cell death. EVSA-T and EFM-192A cells were treated with increasing amounts of GDC-0917 and Debio1143 for 20 hours. Cell viability was determined as described in Experimental procedures.

D. IAP antagonists GDC-0917 and Debio1143 trigger c-IAP1 degradation and p100 processing. A2058 and MDA-MB-231 cells were treated were treated with 1 μM GDC-0197 (G917) or Debio1143 (Debio) for indicated time periods and p100/p52 and c-IAP1 protein levels in cellular lysates were determined by immunoblotting with the indicated antibodies.

Experiments were repeated three times with representatives shown in B and D.

Supplementary Figure 2. Examination of anti-tumor efficacy and tumor to plasma ratio for GDC-017 and Debio1143 in MDA-MB-231-X1.1 model.

A. Individual tumor volume traces for the efficacy study presented in Fig. 1C.

B. Levels of GDC-0917 and Debio1143 in tumors and plasma from the study described in Fig. 1C and partly described in 1D. Experimental description applies from Figure 1.

Supplementary Figure 3. GDC-017 is more efficient then Debio1143 in promoting c-IAP1 degradation.

A. GDC-0917 is more efficient compared to Debio1143 in promoting c-IAP1 degradation in liver. Mice bearing subcutaneous MDA-MB-231-X1.1 xenografts were treated with GDC-0917 (G917), Debio1143 or vehicle as indicated above and euthanized at 4, 8 or 12 hours after their final dose. Liver tissue lysates were prepared as described in

Experimental procedures. Expression of c-IAP1 and 2 was examined by western blotting with the indicated antibody and with GAPDH as a loading control.

B. GDC-0917 is more efficient compared to Debio1143 in promoting c-IAP1 degradation in tumor tissues. Mice bearing subcutaneous LL/2 (LLC1) tumors were treated once with 15 mg/kg of GDC-0917 (G917), or 30 mg/kg of Debio1143, or vehicle and euthanized 4, 8 or 12 hours later. Tumor tissue lysates were prepared as described in Experimental procedures. Expression of c-IAP1 and 2 was examined by western blotting with the indicated antibody and with GAPDH as a loading control.

One of two repeats shown for A and B.

Supplementary Figure 4. Uncropped scans.

Figure S1

A

Compound/KD (nM)	XIAP BIR2	XIAP BIR3	c-IAP1 BIR2	c-IAP1 BIR3
Debio1143	> 30000	11	> 30000	0.065
GDC-0917	> 30000	< 0.80	> 30000	0.47

B

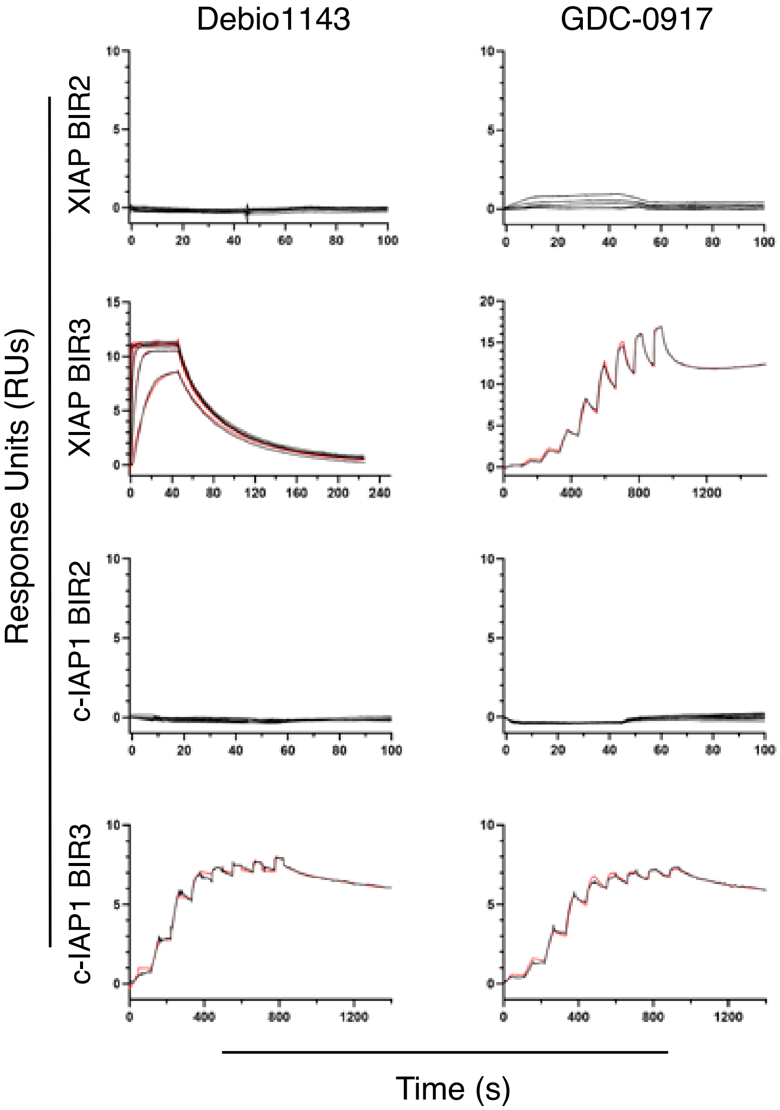

C

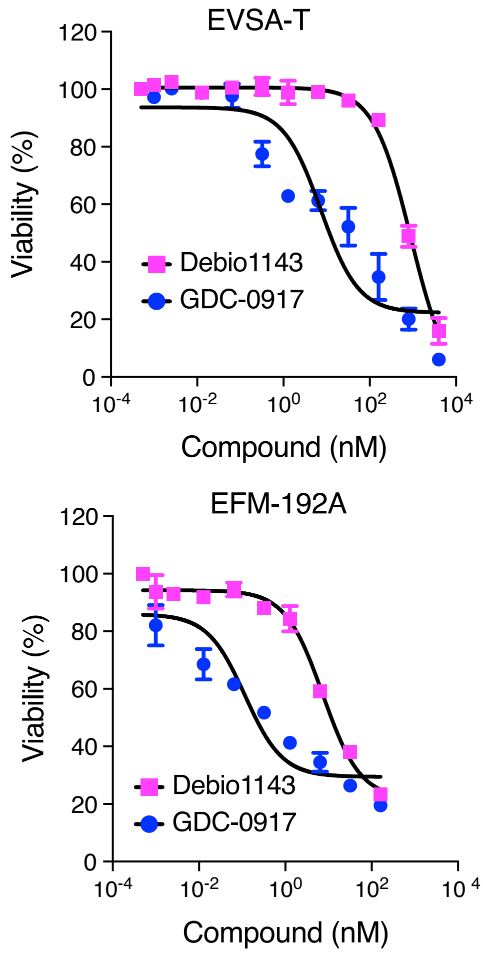

D

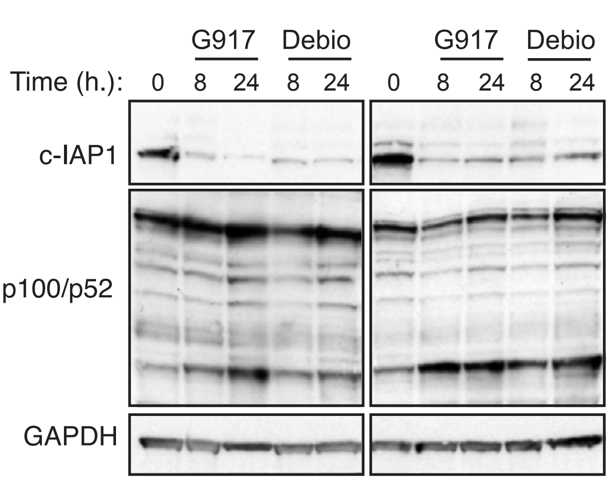

Figure S2

A

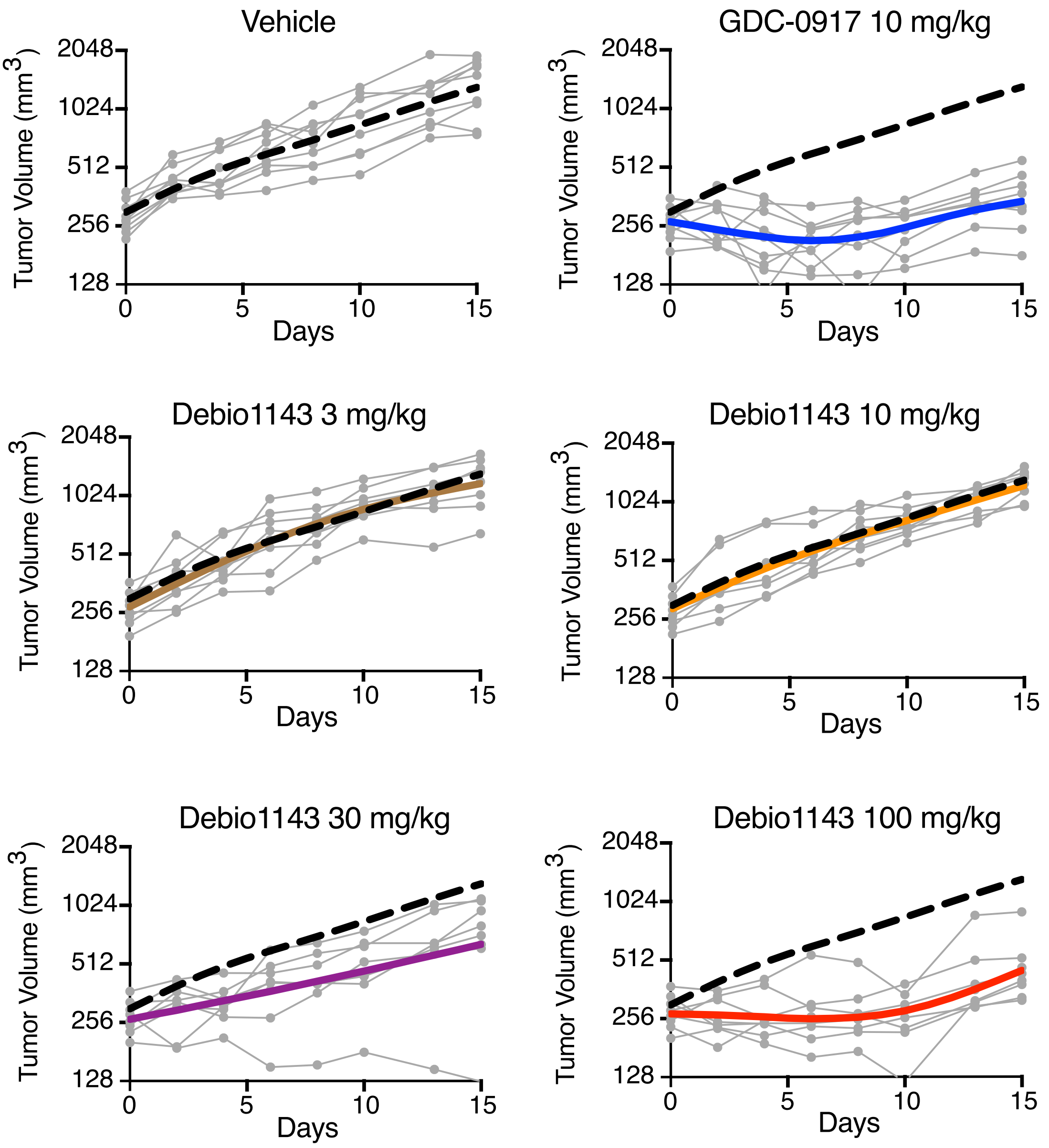
Vehicle
GDC-0917 10 mg/kg
Debio1143 3 mg/kg
Debio1143 10 mg/kg
Debio1143 30 mg/kg
Debio1143 100 mg/kg
Tumor Volume (mm3)
2048
1024
512
256
128
Days
0
5
10
15

B

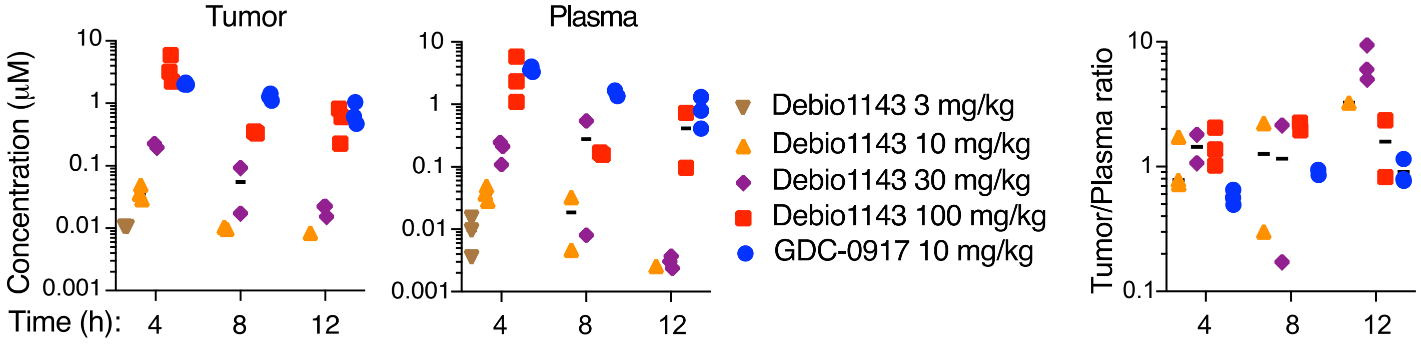
Tumor
Plasma
Concentration (μM)
10
1
0.1
0.01
0.001
Time (h):
4
8
12
Debio1143 3 mg/kg
Debio1143 10 mg/kg
Debio1143 30 mg/kg
Debio1143 100 mg/kg
GDC-0917 10 mg/kg
Tumor/Plasma ratio

Figure S3

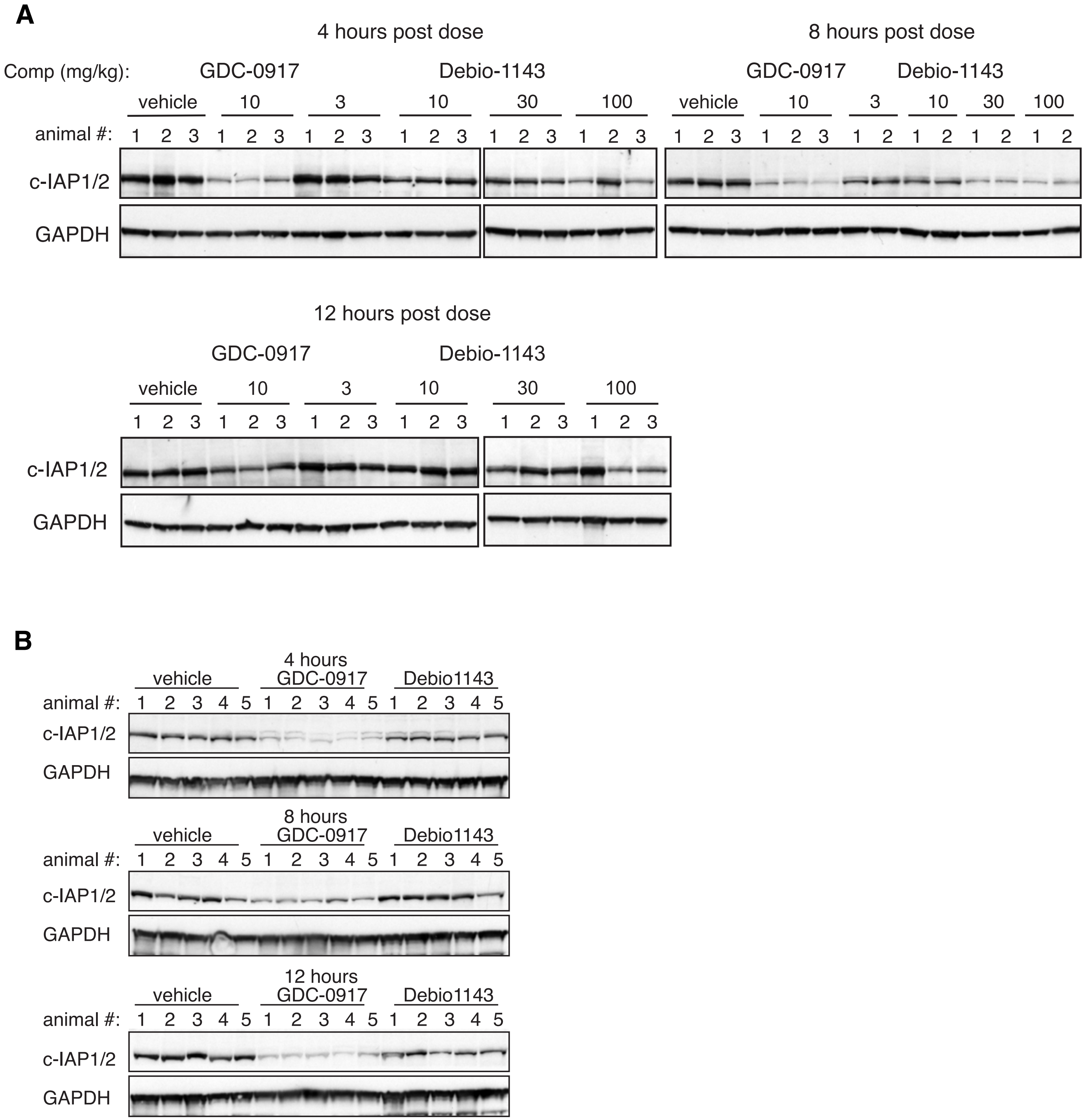

A
4 hours post dose
8 hours post dose
Comp (mg/kg):
GDC-0917
Debio-1143
vehicle
10
3
10
30
100
animal #:
1 2 3
c-IAP1/2
GAPDH
12 hours post dose
B
4 hours
8 hours
12 hours
vehicle
GDC-0917
Debio1143
animal #:
1 2 3 4 5
c-IAP1/2
GAPDH

Figure S4

Fig. 1B

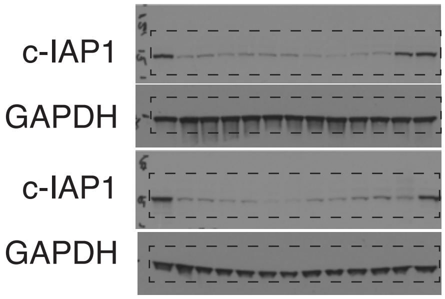

Fig. 1F

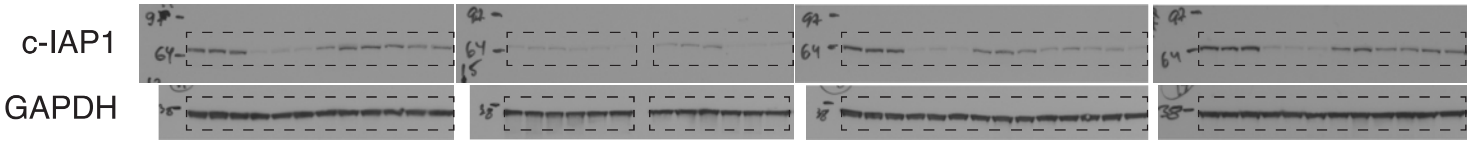

Fig. S1D

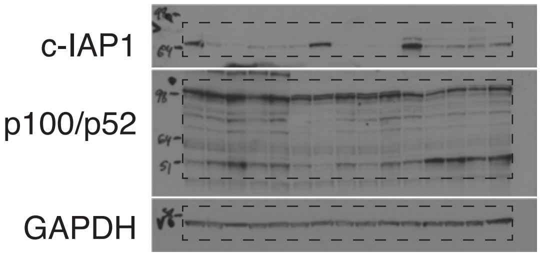

Fig. S3A

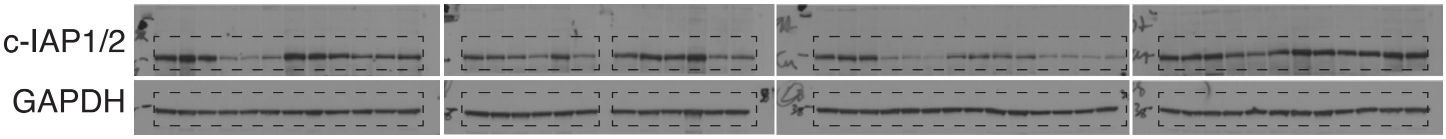

Fig. S3B

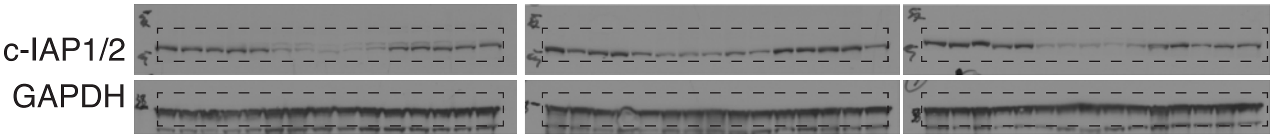

Supplement: Supplementary file 1 — Supplemental Material [file 41419_2022_5283_MOESM1_ESM.pdf]
